# MicroRNAs in pancreatic cancer drug resistance: mechanisms and therapeutic potential

**DOI:** 10.3389/fcell.2024.1499111

**Published:** 2025-01-15

**Authors:** Fangying Dong, Jing Zhou, Yijie Wu, Zhaofeng Gao, Weiwei Li, Zhengwei Song

**Affiliations:** ^1^ Emergency Department, The Second Affiliated Hospital of Jiaxing University, Jiaxing, Zhejiang, China; ^2^ Department of Surgery, The Second Affiliated Hospital of Jiaxing University, Jiaxing, Zhejiang, China; ^3^ Department of general practice, Taozhuang Branch of the First People’s Hospital of Jiashan, Jiaxing, Zhejiang, China

**Keywords:** pancreatic cancer, microRNA, drug resistance, therapeutic targeting, chemoresistance mechanisms

## Abstract

Pancreatic cancer (PC) remains one of the most lethal malignancies, primarily due to its intrinsic resistance to conventional therapies. MicroRNAs (miRNAs), key regulators of gene expression, have been identified as crucial modulators of drug resistance mechanisms in this cancer type. This review synthesizes recent advancements in our understanding of how miRNAs influence treatment efficacy in PC. We have thoroughly summarized and discussed the complex role of miRNA in mediating drug resistance in PC treatment. By highlighting specific miRNAs that are implicated in drug resistance pathways, we provide insights into their functional mechanisms and interactions with key molecular targets. We also explore the potential of miRNA-based strategies as novel therapeutic approaches and diagnostic tools to overcome resistance and improve patient outcomes. Despite promising developments, challenges such as specificity, stability, and effective delivery of miRNA-based therapeutics remain. This review aims to offer a critical perspective on current research and propose future directions for leveraging miRNA-based interventions in the fight against PC.

## 1 Introduction

PC, the most common type being pancreatic adenocarcinoma (PDAC), remains one of the deadliest malignancies, characterized by its aggressive nature, late-stage diagnosis, and resistance to conventional therapies (Cai et al., 2021; Farhangnia et al., 2024; Furukawa, 2009; Neoptolemos et al., 2018; Park et al., 2021; Qin et al., 2020). Despite advances in surgical techniques, radiation, and systemic treatments, including chemotherapy and targeted therapies, the overall survival (OS) rate for PDAC patients remains dismally low, with a 5-year survival rate of less than 10% (Chen K. et al., 2021; Shaib et al., 2006). For the small proportion of patients (approximately 15%–20%) diagnosed at a resectable stage, surgical resection remains the primary treatment modality, offering the best chance for long-term survival (Siegel et al., 2021). However, even after surgery, recurrence rates are high, necessitating the use of adjuvant therapies such as gemcitabinewith with nab-paclitaxel or FOLFIRINOX to improve survival outcomes (Evans et al., 2024). For patients with borderline resectable or locally advanced disease, neoadjuvant chemoradiotherapy is often employed to shrink tumors and potentially enable resection (Eshmuminov et al., 2023; Ghaneh et al., 2023). In contrast, the majority of PDAC cases are diagnosed at an unresectable or metastatic stage, where systemic chemotherapy becomes the mainstay of treatment (Park et al., 2021). Gemcitabine with nab-paclitaxel or FOLFIRINOX are commonly used regimens in the treatment of PDAC. The latter is a combination chemotherapy regimen that includes four drugs: 5-fluorouracil (5-FU), leucovorin, oxaliplatin, and irinotecan. These drugs have different mechanisms of action and work synergistically to enhance treatment efficacy. For example, 5-FU is a pyrimidine analog that interferes with DNA synthesis by inhibiting thymidylate synthase, leading to cell death. However, the overall effectiveness of these therapies is often hampered by significant toxicity and the rapid development of resistance (Shirakawa et al., 2023). Radiotherapy is occasionally employed for local control, but its utility is similarly limited by resistance (Ben-Josef and Lawrence, 2011). The high prevalence of chemoresistance and radioresistance in PDAC underscores the urgent need for novel therapeutic strategies to improve patient outcomes and overcome these barriers (Bear et al., 2020; Tiriac et al., 2018).

Recent research has highlighted the pivotal role of miRNAs, small non-coding RNAs that regulate gene expression at the post-transcriptional level, in mediating drug resistance. miRNAs have emerged as key regulators of various cellular processes, including proliferation, apoptosis, and metastasis, all of which are crucial in the context of cancer therapy (Saliminejad et al., 2019; Sun et al., 2010; Wilczynska and Bushell, 2015). In addition, miRNAs play a crucial role in the regulation of gene expression by acting as post-transcriptional modulators (Lou et al., 2018). In PDAC, miRNA expression is often dysregulated, with some miRNAs being upregulated and others downregulated, contributing to tumorigenesis and disease progression (Madadjim et al., 2024). Dysregulation can result from genetic and epigenetic alterations, such as mutations, DNA methylation, or histone modifications, as well as abnormal transcription factor activity (Baradaran et al., 2019; Pan G. et al., 2021; Singh et al., 2012; Wang et al., 2021). For example, oncogenic miRNAs (oncomiRs) are frequently upregulated in PDAC, promoting tumor growth and chemoresistance by targeting tumor suppressor genes (Fathi et al., 2021). Conversely, tumor-suppressive miRNAs are often downregulated, leading to unchecked activation of oncogenic pathways (Fathi et al., 2021). In PC, abnormal miRNA expression patterns are also related to the regulation of drug resistance mechanisms, affecting the efficacy of chemotherapy drugs and radiotherapy. Previous studies, including a review by Radu et al., have highlighted the potential of circulating miRNAs as non-invasive biomarkers in digestive tract tumors, including PDAC (Albulescu et al., 2011). These miRANs, which are unregulated during tumorigenesis and progression, can reflect the molecular state of tumors and provide insights into drug resistance mechanisms. Targeting specific miRNAs, either to restore tumor suppressor miRNAs or to inhibit carcinogenic miRNAs, can serve as novel therapeutic strategies to overcome PDAC resistance and improve therapeutic outcomes.

Understanding the interplay between miRNAs and drug resistance pathways is essential for developing novel therapeutic strategies and improving treatment outcomes. miRNAs can affect drug resistance through multiple mechanisms, such as the regulation of drug metabolism, modulation of drug targets, and alteration of cellular stress responses (Abo-Al-Ela and Faggio, 2021; Du et al., 2019; Gupta et al., 2021; Khan et al., 2023; Lu and Rothenberg, 2018; Zhang et al., 2019). Moreover, their involvement in the epithelial-to-mesenchymal transition (EMT) (Pan et al., 2021b; Uhan and Hauptman, 2021), a process linked to increased drug resistance, further underscores their relevance in PDAC therapy.

In this review, we aim to provide a comprehensive analysis of the current knowledge regarding the role of miRNAs in PDAC drug resistance. We will explore the mechanistic insights into how specific miRNAs contribute to resistance against various therapeutic modalities, including chemotherapy, targeted therapies, and immunotherapy. Additionally, we will discuss the potential of miRNAs as biomarkers for predicting treatment response and as therapeutic targets to overcome drug resistance. By synthesizing recent findings, this review seeks to enhance our understanding of miRNA-mediated drug resistance and to propose future research directions that could lead to more effective treatment strategies for PDAC.

## 2 The role of miRNAs in PDAC

**FIGURE 1:**
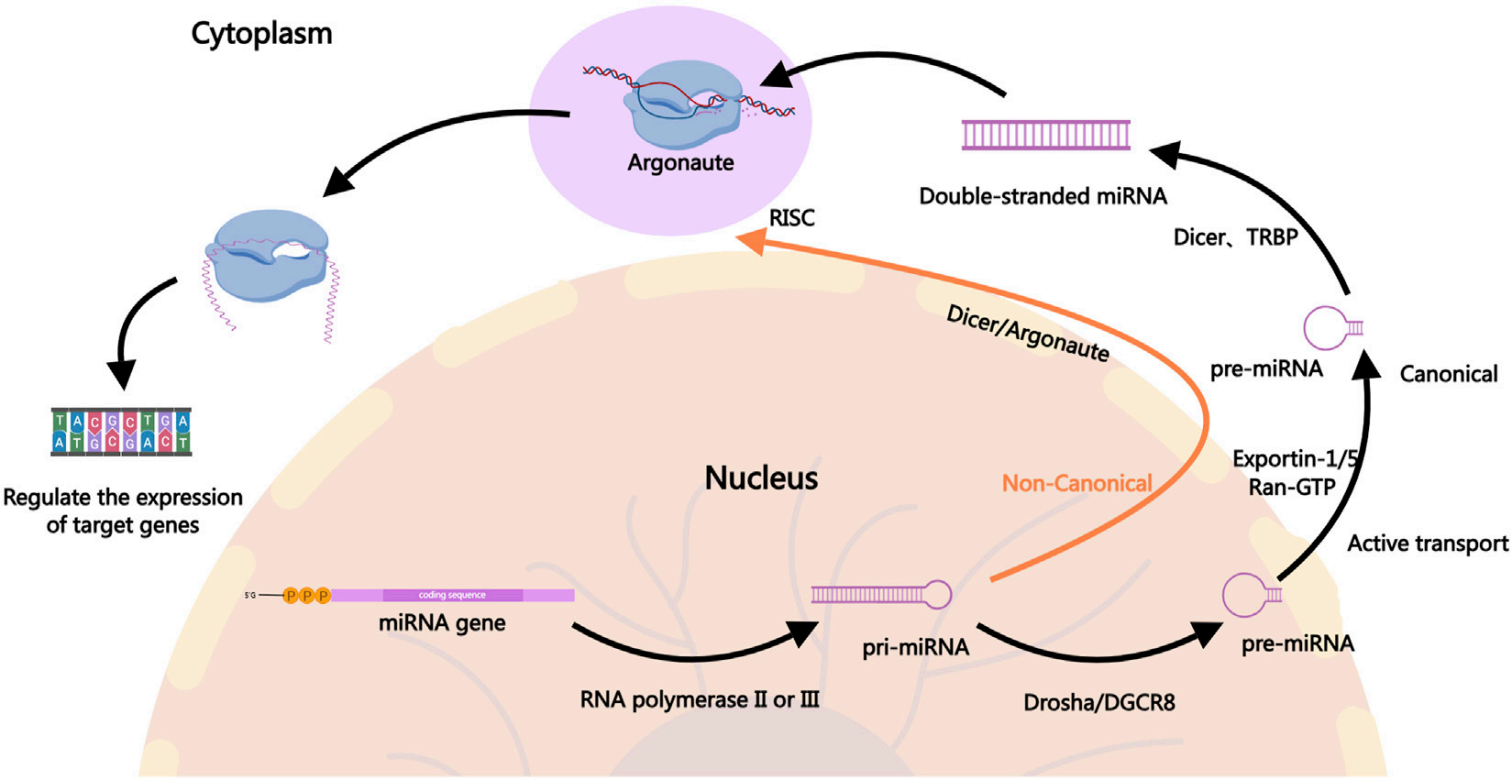
The Biogenesis of miRNAs. In the canonical pathway, miRNA biogenesis begins in the nucleus where pri-miRNAs are transcribed by RNA polymerase II. The pri-miRNAs are then cleaved by the Drosha-DGCR8 complex to form precursor pre-miRNAs, which are exported to the cytoplasm via Exportin-1, Exportin-5, and others. In the cytoplasm, the pre-miRNAs are further processed by Dicer into mature miRNA duplexes. One strand of the duplex, the guide strand, is incorporated into the RISC, where it guides the complex to target mRNAs for silencing or degradation, ultimately regulating gene expression. In the non-canonical pathway, the generation of pri-miRNA is similar to the canonical pathway, but the processing of pri-miRNA into pre-miRNA does not fully depend on Drosha. In the non-canonical pathway, Dicer may directly process incompletely cleaved pri-miRNA or generate pre-miRNA through other enzyme systems, such as Argonaute proteins. In some cases, miRNAs from the non-canonical pathway may be directly cleaved and processed by mediators like Ago2 (a form of Argonaute protein). The mature miRNAs generated through the non-canonical pathway typically enter the RISC in the cytoplasm and participate in gene expression regulation. They may inhibit the translation of target genes or promote their degradation by binding to the 3′UTR of target mRNA.

miRNAs are small non-coding RNAs approximately 22 nucleotides in length that regulate gene expression post-transcriptionally by binding to the 3′untranslated regions (3′UTR) of target mRNAs (Ambros, 2004; Kim and Nam, 2006). In the canonical pathway, the biogenesis of miRNAs involves the transcription of primary miRNA, which is then processed by the Drosha enzyme into precursor miRNA, followed by further cleavage into mature miRNAs by the Dicer enzyme in the cytoplasm (Alarcon et al., 2015; Fellmann et al., 2013; Ha and Kim, 2014; Meister, 2023; Son et al., 2023; Zapletal et al., 2022). Mature miRNAs associate with the RNA-induced silencing complex to bind and regulate target mRNA expression, influencing various cellular processes such as proliferation, apoptosis, and stress responses (Gregory et al., 2005; Iwakawa and Tomari, 2022; Jiang et al., 2019; Lynam-Lennon et al., 2009; Marsit et al., 2006). In the non-canonical pathway, the processing of pri-miRNA is not entirely dependent on Drosha. Different enzymes may be involved in the processing, such as Dicer directly processing incompletely cleaved pri-miRNA or generating pre-miRNA through other enzyme systems, including Argonaute proteins (Ha and Kim, 2014). Figure 1 illustrates the biogenesis of miRNAs.

In PDAC, the expression patterns of miRNAs are significantly altered. These aberrant miRNA expressions are closely linked to key tumor characteristics, including tumor proliferation, metastasis, and drug resistance (Fang et al., 2018; Gu et al., 2022; Liu et al., 2021). For instance, miR-21, miR-155, and miR-210 are highly expressed in PDAC cells, where they promote tumor growth and metastasis by regulating cell cycle-related proteins and anti-apoptotic factors (Abue et al., 2015; Ho et al., 2010; Pang et al., 2015; Sicard et al., 2013). Conversely, the downregulation of miR-34a and miR-143 is associated with increased tumor aggressiveness and poor prognosis in PDAC (Xie et al., 2019; Xin et al., 2019).

miRNAs also play a critical role in the development of drug resistance in PDAC. Drug resistance is a major cause of treatment failure in PDAC and involves complex molecular mechanisms. miRNAs influence drug efficacy through mechanisms such as regulating drug metabolism, modulating drug target expression, and altering cellular stress responses (Pan et al., 2021c). For example, miR-21 enhances resistance to chemotherapeutic agents by inhibiting tumor suppressor genes such as PTEN and RECK (Giovannetti et al., 2010; Park et al., 2009). Additionally, miR-155 and miR-200 are involved in drug resistance by regulating EMT processes and cell cycle-related proteins, affecting drug sensitivity (Li et al., 2009; Patel et al., 2017).

Given the pivotal role of miRNAs in PDAC, researchers have begun to explore their potential as therapeutic targets and biomarkers. Specific modulation of miRNA expression may help overcome drug resistance and improve therapeutic outcomes. Furthermore, miRNA expression profiles could serve as diagnostic and prognostic biomarkers for PDAC, aiding in the development of personalized treatment strategies (Daoud et al., 2019; Ge et al., 2022). In summary, miRNAs play multifaceted roles in the pathogenesis, progression, and drug resistance of PDAC. A deeper understanding of miRNA functions provides crucial insights into the biological characteristics of PDAC and offers important clues for developing novel therapeutic strategies.

## 3 miRNAs in PDAC resistance

**FIGURE 2:**
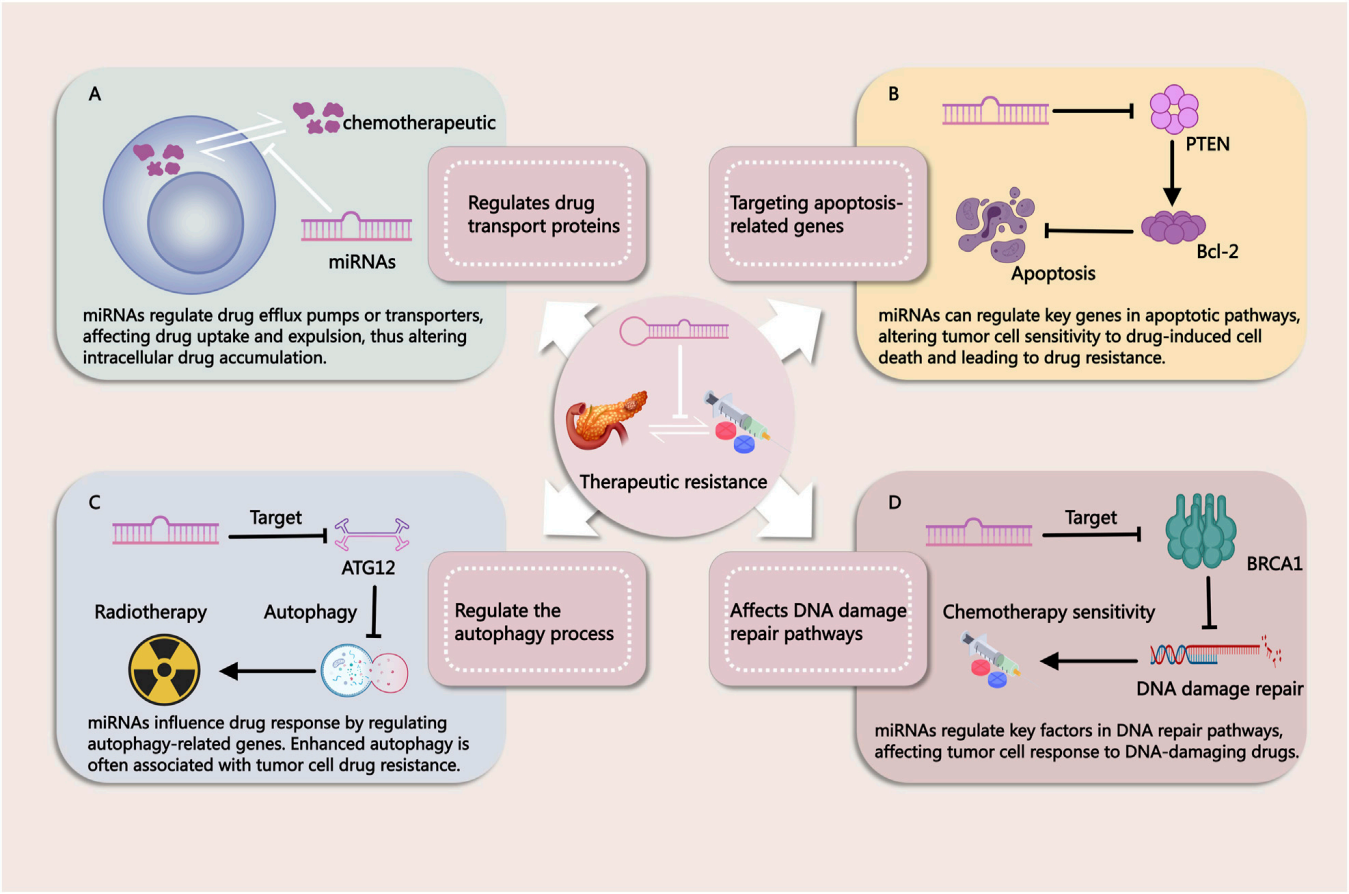
Mechanisms by Which miRNAs Influence Tumor Treatment Resistance. **(A)** miRNAs regulate drug efflux pumps or transport proteins, affecting drug uptake and efflux in cancer cells. This modulation alters intracellular drug accumulation, contributing to treatment resistance. **(B)** miRNAs modulate key genes in apoptotic pathways, thereby influencing the sensitivity of tumor cells to drug-induced cell death. Changes in apoptosis regulation can lead to resistance against chemotherapy. **(C)** miRNAs regulate autophagy-related genes, impacting cellular responses to therapy. Enhanced autophagy is often associated with drug resistance in tumor cells. **(D)** miRNAs influence DNA repair pathways by targeting critical factors involved in DNA damage response. This modulation alters the effectiveness of DNA-damaging agents, contributing to therapy resistance.

miRNAs have emerged as key regulators in the development of resistance to both chemotherapy and radiotherapy in PDAC. Due to their ability to post-transcriptionally regulate multiple genes involved in cell survival, apoptosis, and DNA repair, miRNAs significantly impact the effectiveness of these conventional treatments (Figure 2). Chemotherapy resistance, which limits the success of agents like gemcitabine (GEM), and radiotherapy resistance, which reduces the efficacy of radiation-induced cancer cell death, are both influenced by miRNA-mediated pathways. Understanding these mechanisms is essential for addressing treatment challenges and developing more effective therapeutic strategies to overcome resistance in PDAC.

### 3.1 miRNAs in chemotherapy resistance

Chemotherapy for PDAC primarily relies on drugs such as GEM, 5-FU, and cisplatin, with the aim of shrinking tumors and controlling the disease (Okusaka and Furuse, 2020; Rehman et al., 2022). However, chemoresistance remains a major challenge in treatment, often arising from drug efflux mechanisms, enhanced DNA repair capabilities, suppression of apoptotic pathways, and the complexity of the tumor microenvironment (TME) (Perkhofer et al., 2021; Qian et al., 2019).

Several studies have evaluated the relationship between miR-21 expression and OS in patients with PDAC undergoing GEM treatment. High miR-21 expression has been significantly associated with shorter OS and linked to increased aggressiveness and GEM resistance in PDAC. In both cell and tissue samples, overexpression of miR-21 reduced the antiproliferative effects of GEM and induced apoptosis, while upregulating matrix metalloproteinase (MMP)-2/MMP-9 and vascular endothelial growth factor (VEGF) expression. Inhibition of phosphorylated Akt by phosphoinositide 3-kinase (PI3K) inhibitors and rapamycin blocked pre-miR-21-induced GEM resistance. These findings suggest that miR-21 mediates GEM resistance by regulating apoptosis, Akt phosphorylation, and genes related to invasive behavior, providing mechanistic insights for developing new targeted therapies (Giovannetti et al., 2010). Additionally, other studies have shown that miR-21 confers resistance to 5-FU in PDAC cells by suppressing tumor suppressor genes PTEN and PDCD4. In resistant PDAC cells, miR-21 expression is significantly upregulated, and its inhibition reduces 5-FU resistance. Conversely, overexpression of miR-21 promotes PDAC cell proliferation, migration, and invasion. Restoration of PTEN and PDCD4 expression reverses miR-21-induced resistance and cellular migration, suggesting that miR-21 is a key regulator of 5-FU resistance in PDAC (Wei et al., 2016). Furthermore, miR-21 overexpression has been significantly correlated with shorter disease-free survival (DFS) in patients undergoing GEM adjuvant therapy following radical surgery (Morinaga et al., 2016). Therefore, miR-21 may serve as an important biomarker for predicting GEM resistance in PDAC patients.

In another study, miR-210 was found to be significantly downregulated in GEM-resistant PDAC cells, while its overexpression enhanced GEM sensitivity and exhibited cytotoxic effects on resistant cells. Mechanistically, miR-210 induces caspase-3-mediated apoptosis and inhibits cell proliferation and metastasis by suppressing the expression of ABCC5. A negative correlation between miR-210 and ABCC5 expression was observed in PDAC cell lines and patient tissues, indicating that miR-210 plays a key role in GEM resistance by regulating ABCC5, making it a potential therapeutic target in PDAC. *In vivo* experiments, microRNA-210 transfection almost completely inhibited the growth, proliferation and metastasis of tumor xenografts, and had no obvious side effects on mice (Amponsah et al., 2017). However, another study demonstrated that exosomes derived from GEM-resistant BxR-CSCs promote resistance in GEM-sensitive BxS and PANC-1 PDAC cells by inhibiting GEM-induced cell cycle arrest and apoptosis, and by enhancing cell migration and tubule formation. miR-210 was significantly upregulated in exosomes from BxR-CSCs, and its expression in GEM-treated BxS and PANC-1 cells increased in a dose-dependent manner. Transfection with miR-210 mimics led to biological changes similar to those induced by exosomes, including activation of the mTOR signaling pathway. These findings suggest that exosomes from GEM-resistant PDAC stem cells mediate resistance through miR-210, affecting GEM-sensitive cells’ resistance properties (Yang et al., 2020). Moreover, research has shown that DLEU2L acts as a competing endogenous RNA (ceRNA) for miR-210-3p in GEM resistance in PDAC. DLEU2L is downregulated in PDAC tissues and binds to miR-210-3p. Overexpression of DLEU2L and silencing of miR-210-3p suppress PDAC cell proliferation, migration, and invasion, while promoting apoptosis. These effects are achieved by inhibiting the Warburg effect (aerobic glycolysis) and the AKT/mTOR signaling pathway. Additionally, BRCA2 was identified as a target gene of miR-210-3p, and DLEU2L upregulated BRCA2 through the ceRNA mechanism. Overexpression of DLEU2L and miR-210-3p interference effectively inhibited pancreatic tumor progression, indicating that DLEU2L plays a crucial role in miR-210-3p-mediated GEM resistance. In xenograft models, circ_0013587 overexpression sensitized erlotinib-resistant AsPC-1 cells to erlotinib (Xu F. et al., 2021). Additionally, miRNAs such as let-7b and miR-374b may play significant roles in cisplatin resistance. For example, overexpression of miR-374b can restore cisplatin sensitivity in the cisplatin-resistant BxPC3-R cells (Schreiber et al., 2016; Shao et al., 2015).

Dicer, a crucial enzyme in miRNA biogenesis, plays a vital role in miRNA maturation. Studies have shown that compared to parental PANC-1 cells, Dicer expression is significantly upregulated in GEM-resistant PANC-1/GEM cells, and elevated Dicer levels are associated with an increased risk of PDAC. Inhibition of Dicer significantly reduces GEM resistance in PANC-1/GEM cells, whereas its overexpression enhances resistance. Further research has revealed that the transcription factor Sp1 targets the promoter region of Dicer, and the ERK/Sp1 signaling pathway regulates Dicer expression in PANC-1/GEM cells, correlating with PDAC progression. PANC-1/GEM cells (GEM/shDicer) with stable Dicer knockdown were injected subcutaneously into NOD/SCID mice treated with gemcitabine 3 weeks later. The PANC-1/GEM mice had larger tumors and were more resistant to gemcitabine than the PANC-1 mice. Knocking down Dicer in PANC-1/GEM cells can shorten the cell cycle and inhibit the growth of pancreatic cancer. This suggests that targeting the ERK/Sp1/Dicer pathway could provide a novel therapeutic strategy to overcome GEM resistance in PDAC (Su et al., 2021).

Further studies on miRNA-mediated chemotherapy resistance in PDAC are summarized in Table 1 for a more comprehensive overview.

**TABLE 1:** miRNAs affecting chemotherapy resistance in PC.

MicroRNA	Mechanistic pathway	Treatment effect	Ref
miR-101-3p	MicroRNA-101-3p is significantly downregulated in resistant cells. Its lipid-based mimics can inhibit RRM1 expression and enhance GEM cytotoxicity	Enhance the cytotoxicity of GEM.	Fan et al. (2016)
miR-1179	circ_0087502 regulates TGFBR2 expression by acting as a “sponge” for miR-1179	Promote GEM resistance	Chen et al. (2023)
miR-1227	circ_0013587 reverses resistance by upregulating E-cadherin through the release of miR-1227, which inhibits E-cadherin	Reverse erlotinib resistance	Xu et al. (2021b)
miR-1285	miR-1285 is significantly downregulated in GEM-PC cell lines. *In vitro* experiments demonstrate that miR-1285 inhibits PC cell proliferation, migration, and invasion while increasing sensitivity to GEM. Western blotting analysis reveals that miR-1285 negatively regulates YAP1 protein levels, which, in conjunction with EGFR and β-catenin, modulates the biological behavior of PDAC cells	Potentially reverse GEM resistance	Huang et al. (2017)
miR-137	Dox induces autophagy in PC cells and downregulates miR-137 expression. Overexpression of miR-137 enhances Dox-induced apoptosis and inhibits autophagy. ATG5 is confirmed as a direct target of miR-137, and overexpression of ATG5 can reverse the antitumor effects mediated by miR-137	Enhance doxorubicin sensitivity	Wang et al. (2019)
miR-142-3p	SBF2-AS1 induces GEM resistance in PC by competitively binding to miR-142-3p, thereby inhibiting the expression of TWF1	Mediates GEM resistance	Hua et al. (2019)
miR-145	circ_0036627 increases S100A16 expression by binding to and inhibiting the activity of miR-145. As an oncogenic factor, S100A16 enhances the invasiveness of PC cells and GEM resistance	Promote GEM resistance	Yu et al. (2024)
miR-146a-5p	miR-146a-5p inhibits PDAC cell proliferation and enhances GEM sensitivity by targeting the 3′-UTR of TRAF6. Additionally, miR-146a-5p downregulates the TRAF6/NF-κB p65/P-gp signaling pathway, which regulates PDAC cell growth and chemoresistance	Enhance GEM sensitivity	Meng et al. (2020)
miR-153	miR-153 is downregulated in PC tumor tissues and is associated with poor prognosis. Transfection of miR-153 mimics increases apoptosis in GEM-resistant PC cells, an effect that is reversed by Snail overexpression. miR-153 reverses GEM resistance in PC cells by directly targeting Snail	Reverse GEM resistance	Liu et al. (2017)
miR-17-92	Overexpression of the miR-17-92 cluster reduces CSC self-renewal, tumorigenicity, and chemoresistance by directly targeting multiple components of the NODAL/ACTIVIN/TGF-β1 signaling pathway and inhibiting downstream targets such as p21, p57, and TBX3. Additionally, miR-17-92 overexpression promotes CSC proliferation and leads to their exhaustion by downregulating p21 and p57	Lead to chemoresistance	Cioffi et al. (2015)
miR-181b	miR-181b enhances GEM resistance in PC cells by inhibiting CYLD and activating NF-κB	Enhance GEM resistance	Takiuchi et al. (2013)
miR-198	miR-198 improves GEM resistance in PDAC cells by disrupting the autophagy process through inhibition of VCP.	Improve GEM resistance	Marin-Muller et al. (2023)
miR-301	miR-301 regulates GEM resistance and promotes EMT by downregulating the expression of cadherin 1	Suppress chemotherapy sensitivity	Funamizu et al. (2019)
miR-200b	The expression of miR-200b is positively correlated with chemotherapy sensitivity. Increasing miR-200b expression enhances chemotherapy sensitivity and promotes the reversal of EMT.	Promote chemotherapy sensitivity	Funamizu et al. (2019)
miR-203	miR-203 targets the 3′UTR of SIK1, promoting proliferation, migration, and invasion of PC cells. Restoring SIK1 expression can reverse these effects mediated by miR-203	Mediate GEM resistance	Ren et al. (2016)
miR-205	miR-205 is significantly downregulated in GEM-resistant PC cells. Overexpression of miR-205 inhibits the proliferation of GEM-resistant cells, reduces cancer stem cell (CSC) proliferation, and tumor growth. miR-205 also restores GEM sensitivity in resistant PC cells by decreasing the expression of Nestin and Ki-67	Enhance GEM resistance	Chaudhary et al. (2017)
miR-20a-5p	miR-20a-5p directly targets the 3′UTR of RRM2, thereby inhibiting its expression and overcoming GEM resistance in PC cells	Enhance GEM sensitivity	Lu et al. (2019)
miR-210	miR-210 induces caspase-3-mediated apoptosis and inhibits cell proliferation and migration by suppressing ABCC5 expression	Enhance GEM resistance	Amponsah et al. (2017)
miR-210	Exosomes from GEM-resistant pancreatic CSCs mediate resistance through the transfer of miR-210, affecting GEM-sensitive cells’ resistance characteristics	Affects GEM sensitivity	Yang et al. (2020)
miR-210-3p	DLEU2L overexpression and miR-210-3p inhibition effectively suppress pancreatic tumor progression	Enhance GEM resistance	Xu et al. (2021b)
miR-21	miR-21 confers resistance to 5-FU in PC cells by inhibiting tumor suppressor genes PTEN and PDCD4	Mediates 5-FU resistance	Wei et al. (2016)
miR-21	Overexpression of miR-21 reduces GEM’s antiproliferative effects and induces apoptosis while upregulating MMP-2/MMP-9 and VEGF. Inhibition of phosphorylated Akt using PI3K inhibitors and rapamycin can prevent GEM resistance induced by pre-miR-21	Enhance GEM resistance	Giovannetti et al. (2010)
miR-21	miR-21 overexpression in PC is significantly associated with shorter DFS in patients receiving adjuvant GEM therapy after radical surgery	Predicts GEM resistance	Morinaga et al. (2016)
miR-222-3p	miR-222-3p-containing M2 macrophage-derived exosomes suppress TSC1 and activate the PI3K/AKT/mTOR pathway	Enhances chemotherapy resistance	Guo et al. (2023b)
miR-1207-5p	miR-296-5p targets the pro-apoptotic gene BOK, inhibiting its expression, thus significantly enhancing PC cell invasion and inducing EMT. Additionally, miR-296-5p decreases sensitivity to 5-FU and GEM.	Mediates resistance to 5-FU and GEM.	Okazaki et al. (2020)
miR-29a	miR-29a regulates the Wnt/β-catenin signaling pathway, playing a role in GEM resistance in PC cells	Enhance GEM sensitivity	Nagano et al. (2013)
miR-30a-3p	GEM resistance is associated with the loss of connexin 43 (Cx43) and decreased intercellular communication. Sulforaphane restores these features and increases treatment sensitivity by inhibiting miR-30a-3p. Transfection with miR-30a-3p inhibitors enhances GEM’s bystander effect, Cx43 expression, and cell communication, while miR-30a-3p mimics suppress Cx43 expression	Enhance GEM resistance	Georgikou et al. (2020)
miR-320a	miR-320a is significantly upregulated in 5-FU-resistant PC cells, promoting cell proliferation, invasion, and resistance by binding to the 3′UTR of PDCD4 mRNA and inhibiting PDCD4 expression	Mediates 5-FU resistance	Wang et al. (2016)
miR-331-3p	miR-331-3p activates the Wnt/β-catenin pathway via ST7L, promoting GEM resistance in PC.	Enhance GEM resistance	Zhan et al. (2020)
miR-34a	miR-34a enhances GEM sensitivity in PDAC cells by targeting and inhibiting the Notch 1 signaling pathway, reducing CSC stemness and proliferation	Enhance GEM sensitivity	Pan et al. (2021c)
miR-374b	miR-374b overexpression in resistant BxPC3-r cells restores cisplatin sensitivity to levels close to parental cells	Reverses cisplatin resistance	Schreiber et al. (2016)
miR-378	Glucocorticoids (GCs) induce miR-378 expression and bind to the GC receptor (GR), promoting autophagy and accelerating PDAC progression. DEX and other GCs induce autophagy and EMT, increasing GEM resistance, whereas GR antagonist mifepristone (RU486) can prevent this process	Enhance GEM resistance	Liu et al. (2022)
miR-429	miR-429 enhances GEM sensitivity by regulating PDCD4 expression	Enhance GEM sensitivity	Yu et al. (2017)
miR-455-3p	miR-455-3p plays a crucial role in PC cell proliferation and resistance by targeting TAZ.	Enhance GEM sensitivity	Zhan et al. (2018)
miR-497	NF-κB1 is upregulated in PC and is a direct target of miR-497. Overexpression of miR-497 inhibits NF-κB1, reducing GEM resistance and metastatic potential of pancreatic CSCs	Enhance GEM sensitivity	Yu et al. (2022)
miR-608	miR-608 participates in GEM resistance mechanisms by targeting RRM1 and CDA in PC cells	Enhance GEM sensitivity	Rajabpour et al. (2017)
miR-663a	GEM increases sensitivity to OSI-027 in PC cells by upregulating miR-663a	Enhances sensitivity to OSI-027	Huang et al. (2019)
miR-7	miR-7 expression is reduced in resistant cells. miR-7 regulates senescence by targeting PARP1/NF-κB signaling. Restoring miR-7 expression enhances GEM sensitivity in PC cells	Enhance GEM sensitivity	Ye et al. (2021)
miRNA-let-7b	LIN28B gene expression interference decreases the proliferation ability of CD44+/LIN28B + pancreatic CSCs, leading to cell cycle arrest due to suppression of cyclin D1 expression under the stimulation of miRNA let-7b	Inhibits resistance to cisplatin and GEM.	Shao et al. (2015)
miR-221	Inhibition of miR-221 increases PANC-1 cell sensitivity to treatment	Promotes chemotherapy sensitivity	Tian et al. (2016)
miR-181a/miR-let-7e	—	Mediates hypoxia-induced drug resistance	Al-Sisan et al. (2023)

### 3.2 miRNAs in radiotherapy resistance

miRNAs play a crucial role in regulating PDAC’s sensitivity to radiation therapy. They can modulate various cellular processes, including cell cycle progression, DNA repair, and apoptosis, which are essential for determining the effectiveness of radiation treatment. Specific miRNAs have been identified as key regulators of the radiation response in PDAC cells. Understanding the roles of these miRNAs can provide valuable insights into improving the efficacy of radiation therapy and overcoming resistance in PDAC treatment.

Research has shown that miR-26a is transiently upregulated in PDAC cells following radiation therapy, before returning to normal levels. Although miR-26a is generally classified as a tumor-suppressive miRNA, its transient upregulation significantly promotes radiation resistance, while stable overexpression inhibits it. The transient upregulation of miR-26a enhances radiation resistance by promoting cell cycle arrest and DNA damage repair. miR-26a directly targets the oncogene HMGA2, increasing radiosensitivity, and may also affect PTGS2 by delaying PGE2 synthesis, thereby promoting tumor regeneration (Jiang et al., 2024). Additionally, study has found that miR-23b plays a critical role in pancreatic cancer radiotherapy by regulating the autophagic process. Studies have shown that overexpression of miR-23b inhibits autophagy, enhancing radiosensitivity, whereas miR-23b inhibition promotes autophagy and increases radioresistance. miR-23b exerts its effect by suppressing the expression of the autophagy-related gene ATG12, thereby reducing autophagic flux and influencing the cancer cells’ response to radiotherapy. Therefore, evaluating miR-23b expression levels could serve as a predictive biomarker for radiosensitivity. Autophagy inhibitors, such as chloroquine, can be used in combination to enhance radiosensitivity. Furthermore, delivering miR-23b mimics or inhibitors to modulate autophagy may provide a novel therapeutic approach to improve the efficacy of radiotherapy (Wang et al., 2013a). Ionizing radiation can activate mTOR in PDAC cells by reducing miR-99b expression. miR-99b negatively regulates mTOR expression. The use of the mTOR inhibitor AZD8055 (10 nM, 100 nM, 500 nM) significantly enhances the inhibitory and apoptotic effects of radiation on PDAC cells. In a human PDAC xenograft model, fractionated radiation combined with AZD8055 treatment significantly improved antitumor effects compared to radiation or AZD8055 treatment alone, resulting in a notable reduction in tumor volume. These results suggest that combining AZD8055 with radiation can effectively overcome radiation resistance in PDAC (Wei et al., 2013). Moreover, miR-153 enhances radiation sensitivity by directly targeting the jagged-1 (JAG1) Notch ligand. Adding recombinant JAG1 protein can reverse the therapeutic effects of miR-153 (Zhao et al., 2021).

Above evidence demonstrates that miRNAs play a pivotal role in regulating radiation therapy resistance in PDAC. They influence critical processes such as cell cycle progression, DNA repair, and apoptosis, highlighting their potential as targets to enhance treatment outcomes and address resistance.

### 3.3 The role of the TME in therapeutic resistance in PDAC and the involvement of miRNAs

The TME plays a pivotal role in modulating the therapeutic response in PDAC (Sherman and Beatty, 2023). The TME consists of a complex network of stromal cells, immune cells, extracellular matrix components, and signaling molecules that interact with tumor cells, thereby influencing tumor progression and resistance to treatment (Werba et al., 2023). In PDAC, the TME is often characterized by a dense stroma, consisting of cancer-associated fibroblasts (CAFs), myofibroblasts, and extracellular matrix proteins, which contribute to tumor progression and treatment resistance (Werba et al., 2023). Furthermore, the TME is rich in inflammatory cytokines, growth factors, and hypoxic regions, all of which create a hostile environment that protects the tumor from conventional therapies such as chemotherapy, radiotherapy, and immunotherapy (Ho et al., 2020).

CAFs, which are a major component of the PDAC stroma, promote tumor progression by secreting cytokines, growth factors, and extracellular matrix components that not only facilitate tumor cell survival but also contribute to drug resistance (Yamashita and Kumamoto, 2024). They create a physical barrier that limits drug delivery and reduce the efficacy of chemotherapies (Ferrara et al., 2021). Additionally, CAFs secrete exosomes that carry signaling molecules, including miRNAs, which can influence the behavior of neighboring cells within the TME, such as immune cells and tumor cells (Qi et al., 2023).

Qi et al. have shown that CAFs-derived exosomes in PDAC do not inherently promote GEM resistance under normal conditions. However, after exposure to GEM, CAFs may undergo changes that enhance their ability to contribute to GEM resistance. After GEM treatment, CAFs promote chemotherapy resistance of PDAC cells by secreting exosomes and maintaining signal communication with cancer cells. Mechanistically, miR-3173-5p from CAF exosomes sponges ACSL4 and inhibits ferroptosis after uptake by cancer cells (Qi et al., 2023). In addition, other study has shown that GEM can upregulate the expression of Snail and miR-146a in PDAC CAFs exosomes, thereby increasing the proliferation and survival of epithelial cells, and Snail and miR-146a can be delivered to cells through CAF exosomes (Richards et al., 2017).CAF-derived exosomes can also regulate the levels and functions of miR-125b-5p, PTEN-targeting miRNAs (including miR-21, miR-181a, miR-221, miR-222, and miR-92a), and miR-224-3p, thereby promoting the progression and drug resistance of PDAC (Guo et al., 2023a; Richards et al., 2022; Zhang et al., 2024). However, there is no evidence that these CAF-controlled miRNAs can regulate the activity of immune cells in PDAC.

The immune cells within the TME, including tumor-associated macrophages (TAMs), also play significant roles in mediating therapeutic resistance (Khalaf et al., 2021). TAMs, which are often polarized into the pro-tumorigenic M2 phenotype, promote tumor growth, angiogenesis, and immune suppression (Pan et al., 2020). miRNAs have been shown to regulate TAM polarization and activity, thereby influencing the immune landscape within the tumor. For instance, a study has shown that TAMs contribute to gemcitabine resistance in PDAC through the secretion of exosomes containing miR-365. These exosomes transfer miR-365 to cancer cells, leading to gemcitabine inactivation by upregulating the triphospho-nucleotide pool and inducing cytidine deaminase. Blocking miR-365 or inhibiting exosome secretion enhances gemcitabine sensitivity, highlighting TAM-derived miR-365 as a key regulator of drug resistance and a potential therapeutic target (Binenbaum et al., 2018).

Moreover, hypoxia, a common feature of the PDAC microenvironment, has been linked to the upregulation of specific miRNAs that contribute to tumor progression and drug resistance (Chen et al., 2024). Under hypoxic conditions, HIF-1α influences the expression of MKLN1-AS by directly binding to anoxic response elements in the MKLN1-AS promoter region. By binding to miR-185-5p as a competitive endogenous RNA, MKLN1-AS regulates the expression of TEAD1 and promotes cell proliferation, migration and tumor growth. TEAD1 then facilitated the development of PDAC (Chen et al., 2024).

In addition to immune cells and stromal cells, extracellular matrix components, such as fibronectin and collagen, interact with integrins on tumor cells, facilitating survival and drug resistance (Jiang et al., 2022). miRNAs can regulate integrin expression, further influencing the ability of PDAC tumors to resist therapy. For example, it has been shown that MiR-760 enhances the sensitivity of pancreatic cancer cells to gemcitabine by regulating integrin β1 (Yang et al., 2019).

Above evidence demonstrates that the TME in PDAC plays a crucial role in shaping the response to therapy by promoting resistance through stromal cell signaling, immune modulation, and extracellular matrix remodeling. miRNAs are key players in regulating these processes, influencing the interactions between tumor cells and their microenvironment.

## 4 Targeting miRNAs to combat treatment resistance in PDAC

Current studies have shown that miRNAs have become key regulatory factors in the development of drug resistance in PDAC, with multiple pathways affecting tumor progression. Identifying specific miRNA targets provides a promising approach to overcoming drug resistance in PDAC. By targeting these miRNAs, it is possible to modulate key signaling pathways, enhance therapeutic sensitivity, and improve the overall effectiveness of current therapies.

Studies have shown that DNA methyltransferase DNMT1 suppresses miR-34a expression by hypermethylating the miR-34a promoter, thereby enhancing the activation of the Notch signaling pathway. In PDAC patients, DNMT1 expression is negatively correlated with miR-34a levels. Knockdown of DNMT1 reduces miR-34a promoter methylation, increases miR-34a expression, and inhibits Notch pathway activation. By modulating the DNMT1/miR-34a axis to downregulate the Notch pathway, PDAC cells exhibit significantly enhanced sensitivity to molecular targeted therapies. Hence, indirectly enhancing miR-34a expression by targeting DNMT may be a potential therapeutic target for pancreatic cancer (Ma et al., 2020).

Additionally, it has been discovered that PDAC cells contain a side population (SP) with stem cell-like properties, capable of inducing rapid and aggressive tumor formation in nude mice. The expression of miR-21 and miR-221 differs significantly between SP and non-side population (NSP) cells. Intervening in SP cells with antisense oligonucleotides (ASOs) against miR-21 and miR-221 significantly reduces the proportion of SP cells, impairs their differentiation and downstream gene regulation, and increases their sensitivity to chemotherapy agents GEM and 5-FU. Combined treatment with miR-21 and miR-221 ASOs is more effective than individual treatments, markedly inhibiting primary tumor growth and metastasis, especially in the L3.6 pl (Gres)-SP pancreatic tumor model. This suggests that targeting miR-21 and miR-221 may be a promising strategy for addressing stem cell-like subpopulations in PDAC (Zhao et al., 2015).

Moreover, miR-23B has been identified as an effective autophagy inhibitor. By targeting the 3′UTR of autophagy-related gene ATG12, it reduces autophagy activity, thereby enhancing radiation-induced cell death in PDAC cells. The use of miR-23B mimics or ATG12 inhibitors significantly enhanced the radiotherapy sensitivity of PDAC. Given that PDAC cells exhibit high basal autophagy levels, which may contribute to treatment resistance, miR-23B’s ability to inhibit autophagy suggests that it could improve the therapeutic response in PDAC, providing a potential avenue for optimizing treatment strategies (Wang et al., 2013b).

Furthermore, research has shown that the tumor suppressor p53 regulates the expression of GPR55 through miR-34b-3p. Inhibition of GPR55 decreases anchorage-dependent and -independent growth, cell cycle progression, MAPK pathway activation, and ribonucleotide reductase levels in PDAC cells. GPR55 knockdown also reduces tumor cell proliferation and MAPK pathway activity in KPC mice. *In vivo*, GPR55 gene deletion significantly extends the survival of KPC mouse models. Notably, combining the GPR55 antagonist cannabidiol (CBD) with the chemotherapeutic agent GEM extends the survival of KPC mice nearly threefold compared to GEM alone. These findings suggest that the combination of CBD and GEM may offer a novel therapeutic approach to improve the prognosis of PDAC patients (Ferro et al., 2018).

miR-30a-5p is downregulated in PDAC and is associated with poor prognosis. Upregulation of miR-30a-5p inhibits tumor cell proliferation, increases apoptosis, and significantly enhances sensitivity to GEM chemotherapy. In GEM-resistant PDAC cells, miR-30a-5p expression is markedly reduced. Further investigation identified FOXD1 as a direct target of miR-30a-5p, suggesting that the miR-30a-5p/FOXD1/ERK axis plays a crucial role in the development of GEM resistance in PDAC. Upregulation of its expression with miR-30a-5p mimics can enhance the sensitivity of PDAC to GEM, making it a potential therapeutic target to overcome GEM resistance (Zhou et al., 2019).

We summarize additional relevant targets in Table 2 for a more intuitive presentation. To clinically validate the therapeutic potential of miRNA targeting in pancreatic cancer, a promising approach could involve the use of *in situ* hybridization (ISH) analysis to detect specific miRNAs in patient tissues (Chu et al., 2019; Volpi et al., 2018). ISH offers a powerful method for visualizing miRNA expression patterns directly in the context of the tumor microenvironment, providing insights into how miRNAs are distributed and potentially linked to therapeutic resistance mechanisms (Chu et al., 2019; Volpi et al., 2018). Given the heterogeneity of pancreatic ductal adenocarcinoma (PDAC), ISH could be particularly valuable in identifying miRNA signatures that correlate with treatment responses or resistance in clinical samples. Moreover, it could help pinpoint miRNAs involved in tumor progression, metastasis, and the modulation of the tumor immune microenvironment, all of which are key factors in drug resistance. For example, previous studies have shown that locked nucleic acid-ISH analysis of miR-21 may be an important predictor of gemcitabine resistance in pancreatic cancer patients receiving adjuvant gemcitabine therapy after therapeutic resection (Morinaga et al., 2016).

**TABLE 2:** Key miRNA targets for overcoming drug resistance in PC.

Targets	Mechanistic pathway	Methods of modulation/targeting	Treatment effect	Ref
miR-100	Increased expression in metastatic PDAC cell lines; inhibitors reduce IGF1-R expression	The miR-138 mimics PreMir-138 and the miR-100 inhibitor MirZip-100 were directly regulated	Potential new target for PDAC treatment	Huang et al. (2013)
miR-1246	Regulates chemoresistance and cancer stem cell properties in PDAC through CCNG2	Expression was directly regulated using the Lentiviral pLenti-III-miR-1246 vector	May serve as a new prognostic and therapeutic target.	Hasegawa et al. (2014)
miR-34a	High expression of DNMT1 is negatively correlated with low expression of miR-34a. DNMT1 knockdown reduces miR-34a promoter methylation, increases miR-34a expression, and inhibits Notch pathway activation	Indirectly enhancing miR-34a expression by targeting DNMT	Modulating the DNMT1/miR-34a axis to downregulate the Notch pathway can significantly enhance PDAC cell sensitivity to molecular targeted therapies	Ma et al. (2020)
miR-221-3p	Promotes PDAC cell proliferation, migration, invasion, and drug resistance while inhibiting apoptosis	miR-221-3p mimic or miR-221-3p inhibitor was used for direct regulation	Potential new target for PDAC treatment	Wu et al. (2020)
miR23B	Acts as an effective autophagy inhibitor by targeting the 3′UTR of the autophagy-related gene ATG12, decreasing autophagy activity and enhancing radiation-induced PDAC cell death	Direct or indirect regulation using miR-23B mimics or ATG12 inhibitors	May improve PDAC treatment response by inhibiting autophagy	Wang et al. (2013b)
miR-21	Significant differences in expression between SP and NSP cells. Antisense ASOs targeting miR-21 and miR-221 significantly reduce SP cell proportion, impair SP cell differentiation and downstream gene regulation, and increase sensitivity to GEM and 5-FU.	Antisense ASOs targeting miR-21 and miR-221 was used for direct regulation	Combined treatment with miRNA-21 and miRNA-221 ASOs shows superior efficacy compared to single-agent therapy	Zhao et al. (2015)
miR-345	Overexpression inhibits PDAC cell growth and induces apoptosis, associated with mitochondrial membrane potential loss, cytochrome c release, and activation of caspase-3/7	Direct overexpression of miR-345 was transfected using plasmid vectors	Restoration of miR-345 may provide new directions for PDAC treatment	Srivastava et al. (2015)
Dysregulated miRNA Clusters	Interact with key cancer-related pathways such as AGE-RAGE signaling, prolactin signaling, and insulin resistance signaling	—	These miRNAs hold promise as biomarkers for PC, aiding in understanding disease initiation and progression	Chhatriya et al. (2019)
miR-34b-3p	Regulating GPR55 expression through miR-34b-3p. GPR55 antagonist CBD combined with chemotherapy drug GEM extends survival in KPC mice nearly threefold compared to GEM alone	The tumor suppressor p53 was used to indirectly regulate miR34b-3p	Combined treatment with CBD and GEM offers a promising new strategy to improve prognosis in PDAC patients	Ferro et al. (2018)
miR-216b	Both 5′phosphate and non-phosphate variants effectively inhibit oncogenic KRAS in PDAC cells and suppress colony formation	Single-chain miR-216b mimics were designed for direct regulation	Potential new target for PDAC treatment	Ferino et al. (2018)
miR-137	Downregulated in a time-dependent manner after anoikis induction; promotes PDAC cell apoptosis, with PXN enhancing AKT signaling involved in apoptosis	The expression was directly regulated using siRNA or miR-137 mimics	Plays an important role in anoikis; may become a potential target for PDAC detection and treatment	Li et al. (2020)
miR-760	Targets RNA-binding protein MOV10, reducing MOV10 protein levels and destabilizing ITGB1. Upregulation of ITGB1 can restore cell viability and GEM resistance suppression caused by miR-760 overexpression	Upregulation of ITGB1 inhibited miR-760 expression	Plays a crucial role in enhancing PDAC cell sensitivity to GEM.	Yang et al. (2019)
miR-132	Directly targets TGF-β2 and enhances its expression by binding to its 3′UTR. DEX inhibits miR-132 expression via promoter methylation, increasing TGF-β2 levels. miR-132 mimics reduce DEX-induced vimentin and E-cadherin clonogenicity, migration, and expression	Dexamethasone was used to indirectly regulate miR-132 expression	Key in PDAC progression, providing a basis for miRNA-based therapeutic strategies	Abukiwan et al. (2019)
miR-30a-5p	Upregulation inhibits tumor cell proliferation, increases apoptosis, and significantly enhances sensitivity to GEM, potentially related to the miR-30a-5p/FOXD1/ERK axis	Direct regulation using miR-30a-5p mimics	May be a potential therapeutic target for overcoming GEM resistance	Zhou et al. (2019)
miRNAs Associated with Carcinogenic Signaling Pathways	Enhances GEM sensitivity in PDAC cells through specific miRNA biomarkers, reducing drug resistance	—	Combination improves treatment efficacy in PaCa patients	Parasramka et al. (2013)

## 5 Summary and future directions

In summary, miRNAs have emerged as pivotal regulators in the context of drug resistance in PDAC, a malignancy notorious for its poor prognosis and limited treatment options. Our review has comprehensively analyzed the role of miRNAs in modulating the effectiveness of conventional therapies, including GEM, 5-FU, oxaliplatin and radiotherapy, by influencing key mechanisms such as drug metabolism, cellular apoptosis, and DNA repair pathways. We have elucidated how specific miRNAs contribute to resistance by targeting critical signaling pathways and have discussed the potential of leveraging these molecules for therapeutic and diagnostic purposes.

The potential of miRNA-based strategies as innovative tools for overcoming drug resistance is promising, with advances in miRNA profiling and targeted delivery systems offering new avenues for intervention. However, several challenges must be addressed to translate these findings into clinical practice. Key issues include ensuring the specificity of miRNA-targeted therapies, improving the stability and bioavailability of miRNA-based drugs, and developing efficient delivery systems to enhance therapeutic efficacy.

Recent studies have highlighted the critical role of miRNAs in mediating the response of PDAC to radiotherapy. Dysregulation of miRNAs can contribute to the development of radiotherapy resistance by modulating various cellular processes, including autophagy, apoptosis, and DNA damage repair. For example, miR-23b has been shown to inhibit autophagy by targeting the autophagy-related gene ATG12, which in turn enhances radiosensitivity in PDAC cells. Conversely, miR-23b inhibition promotes autophagy and confers radioresistance (Wang et al., 2013b). The interplay between miRNAs and autophagy, as well as their regulation of key cellular pathways involved in the DNA damage response, represents a promising area of research to better understand the mechanisms underlying radiotherapy resistance. Given their ability to modulate multiple molecular targets, miRNAs offer potential as both biomarkers for predicting treatment response and as therapeutic targets to overcome radiotherapy resistance in PDAC. Further investigation into the miRNA-mediated regulation of radioresistance in PDAC will provide critical insights into developing more effective and personalized treatment strategies.

Future research should focus on the following areas: identifying and validating novel miRNAs and their targets involved in drug resistance, developing advanced delivery systems that can precisely target PDAC cells while minimizing off-target effects and exploring combinatorial approaches that integrate miRNA-based therapies with existing treatments to enhance overall efficacy. Additionally, further investigation into the role of miRNAs in resistance mechanisms across different stages of PDAC and their interactions with the TME could provide deeper insights and facilitate the development of more effective therapeutic strategies.

Coupling ISH with tissue microarrays or multiplex staining could enhance its diagnostic utility, allowing for the simultaneous analysis of miRNA expression alongside other biomarkers that contribute to therapy resistance (Eckstein et al., 2019; Rotman et al., 2020). The integration of these techniques could potentially inform the development of personalized miRNA-based therapies, guiding treatment decisions based on individual miRNA profiles and their role in resistance pathways. Furthermore, advancements in RNA sequencing technologies, such as single-cell RNA sequencing, could complement ISH by providing a more comprehensive analysis of miRNA expression at the single-cell level, thereby offering deeper insights into the functional role of specific miRNAs in PDAC (Chen X. et al., 2021; Ligorio et al., 2019). While these approaches are still in the early stages of exploration, their clinical application could pave the way for miRNA-based diagnostic tools and therapies, which could ultimately improve the treatment outcomes of PDAC patients.

It is worth mentioning that, currently, there is limited research on the role of miRNAs in immune therapy resistance specifically in PDAC. However, substantial evidence exists from studies in other cancer types (Romano and Kwong, 2018; Yang et al., 2023). In various malignancies, miRNAs have been found to significantly influence immune therapy outcomes by modulating immune evasion mechanisms, immune cell infiltration, and the expression of immune checkpoint proteins (Kousar et al., 2022; Tang et al., 2022). For instance, miRNAs can impact the effectiveness of immune checkpoint inhibitors by regulating the expression of PD-L1 or affecting T cell activation (Hong et al., 2020; Zhang et al., 2020; Zheng et al., 2023). These insights highlight the potential of exploring miRNA-based strategies to overcome immune therapy resistance in PDAC, offering new avenues for enhancing treatment efficacy.

By addressing these challenges and exploring these future directions, we can advance the field of miRNA-based therapies and potentially improve the clinical management of PDAC, ultimately enhancing patient outcomes and survival rates.

## Abbreviations

MicroRNAs, miRNAs; Pancreatic cancer, PC; Epithelial-to-mesenchymal transition, EMT; 3′untranslated regions, 3′UTR; Overall survival, OS; Pancreatic ductal adenocarcinoma, PDAC; Metalloproteinase, MMP; Vascular endothelial growth factor, VEGF; Phosphoinositide 3-kinase, PI3K; 5-fluorouracil, 5-FU; Tumor microenvironment, TME; Cancer-associated fibroblasts, CAFs; Tumor-associated macrophages, TAMs; Disease-free survival, DFS; Competing endogenous RNA, ceRNA; Gemcitabine, GEM; Cancer stem cell, CSC; Connexin 43, Cx43; Glucocorticoids, GCs; Jagged-1, JAG1; Side population, SP; Non-side population, NSP; Antisense oligonucleotides, ASOs; Cannabidiol, CBD.
